# Acid−Base Flow Batteries for Sustainable Energy Storage: Balancing Energy Recovery and Efficiency

**DOI:** 10.1021/acsomega.5c06024

**Published:** 2025-11-28

**Authors:** Marta Herrero-Gonzalez, Nadia El Arroubi, María Fresnedo San-Roman, Raquel Ibañez

**Affiliations:** Dpto. Ingenierias Química y Biomolecular, ETSIIT, 16761 Universidad de Cantabria, Avda. de los Castros 46, Santander 39005, Spain

## Abstract

Acid−base flow batteries (ABFBs) represent a novel approach to addressing the needs for advanced energy storage solutions to overcome the stochastic nature of renewable generation and ensure a consistent matching of energy supply with demand. ABFB relies on bipolar membrane electrodialysis (BMED) and reverse electrodialysis (BMRED) to generate pH gradient in the charging phase (BMED) through external electric power and then convert it into electricity during the discharging phase (BMRED). Although ABFB could be an ecofriendly and affordable alternative to other commercial batteries, it demands research to increase its current low maturity. This study examines the performance of ABFBs by evaluating operating conditions to maximize the Round-Trip Efficiency (RTE) and Gross Energy Density (GED). Furthermore, the trade-offs between RTE and GED indicators are analyzed for the first time in the literature. Experimental results ascertain that the RTE is maximized with low charging and high discharging current densities, attaining a maximum RTE of 12.6%. The maximum GED is achieved with high current densities for both phases, reaching a maximum of 4.2 Wh/L. Thus, RTE and GED cannot be maximized simultaneously. The indicator to be prioritized should be based on the specific needs of the desired application.

## Graphical abstract

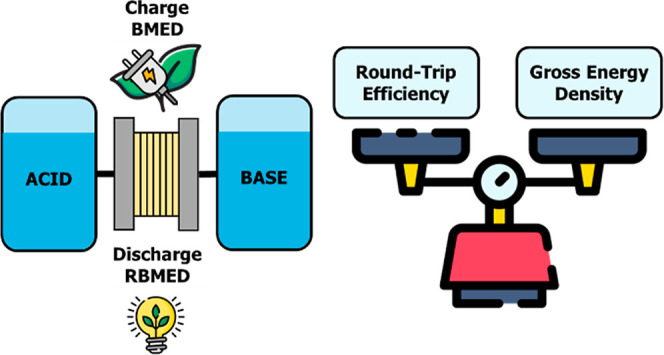

## Introduction

Efforts to achieve affordable and clean energy rely on the development and deployment of renewable energy sources to replace conventional fossil fuel-based systems.^1^ However, this transition faces the significant challenge of overcoming the stochastic nature of renewable generation and ensuring a consistent matching of the energy supply with demand. Advanced storage technologies and grid management solutions are therefore required to ensure a stable and reliable energy supply.^2^ The current state of large-scale energy storage presents certain environmental challenges, including the necessity for materials designated as critical.^3^ Batteries are electrochemical energy storage devices that are increasingly gaining attention due to their high flexibility and ability to be installed in a wide range of locations and applications.^4^

Acid−base flow batteries (ABFBs) represent a novel proposal for electrical energy storage as rechargeable and reusable secondary batteries.^5^ The principle of ABFB involves two clearly differentiated processes, namely, charging phase (Figure 1a) and discharging phase (Figure 1b), based on bipolar membrane electrodialysis (BMED) and bipolar membrane reverse electrodialysis (BMRED) technologies, respectively. Both ABFB phases can be operated in a single conventional BMED module, which is typically assembled with a series of triplets or repeating units comprising anion exchange membranes (AEMs), cation exchange membranes (CEMs), and bipolar membranes (BPMs), all of them separated by means of spacers, creating distinct compartments for the solutions. The membrane stack is completed by integrating electrodes on either side. The electrode material must exhibit chemical compatibility with the employed electrode rinse solution (ERS) and support both reduction and oxidation reactions, as these occur concurrently at each electrode. To mitigate overpotential effects, an ERS containing a reversible redox couple is recommended.

**Figure 1:**
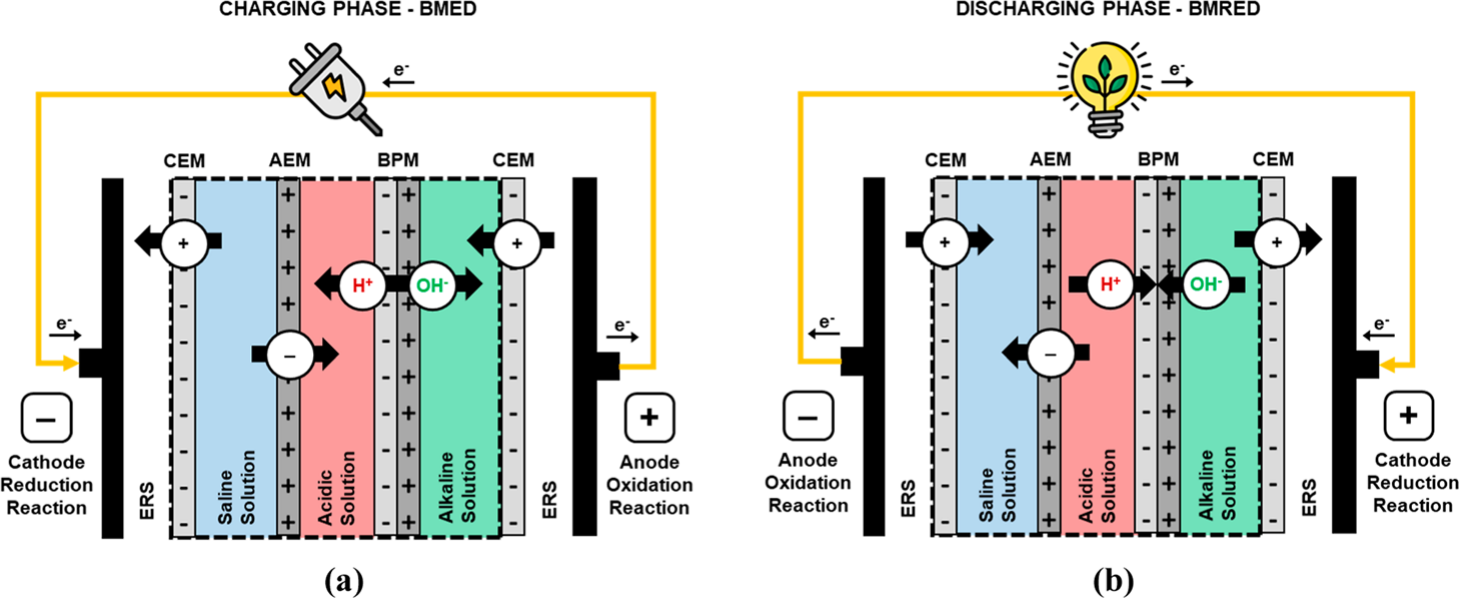
Working principles of ABFB: (a) charging phase (BMED) and (b) discharging phase (BMRED). Dashed square represents the triplet.

In the charging phase (Figure 1a), the application of an electric field generates: (i) the dissociation of water molecules into protons (H^+^) and hydroxide ions (OH^−^) at the BPM interface and (ii) the selective transport of anions and cations through the AEM and CEM, respectively, from the saline solution compartment to the acid and base compartments. During this step, the acidic and alkaline solutions are concentrated, thereby generating a pH gradient, while the concentration of the feed saline solution is reduced. Therefore, the use of renewable energy as the source of electricity presents a substantial opportunity for renewable energy storage. The surplus renewable energy generated during production peaks can be employed to create the requisite pH gradient via BMED. The integration of renewable energy, particularly solar photovoltaics, into the BMED process has been previously explored, revealing that the production of acids and bases is not affected by the source of electricity used, as long as the average current density remains the same.^6–8^

During the discharging phase (Figure 1b), or BMRED, anions and cations are transported from the product compartments to the saline compartment, while protons and hydroxide ions are neutralized at the BPM interlayer, releasing water. Consequently, the concentrations of the acidic and alkaline solutions decrease, while the concentration of the saline compartment increases. The transport of ions, in conjunction with the neutralization process and the redox reaction in the electrodes, generates electron movement and thus an electric current.

Advances in membrane technology and their commercial availability, combined with the growing need for sustainable energy storage systems, have led to renewed interest in ABFBs, prompting a revisitation of the topic. Various aspects of the technology have been investigated, all aiming to maximize energy recovery during the discharge phase and thereby improve the overall efficiency, typically assessed through the Round-Trip Efficiency (RTE), which is defined as the ratio between the energy recovered during the discharging phase and the energy supplied during the charging phase. Among these different aspects, experimental works have focused on: (a) ERS selection to reduce electrode compartment overpotentials,^9^ (b) operating parameters such as charging and discharging current densities,^10^ (c) system performance under multiple cycles,^11,12^ (d) stacking performance by increasing the number of triplets,^13^ (e) solution composition and concentration,^12,14,15^ (f) strategies to minimize losses caused by the occurrence of nonideal phenomena such as parasitic or shunt currents,^16^ and (g) fabrication or modification of membranes.^17^ Other studies have focused on mathematical modeling of the process, aiming to optimize operating conditions and maximize the Round-Trip Efficiency (RTE),^18^ or 3D modeling of the stack for current−voltage behavior prediction and shunt current characterization.^19^ Additionally, ABFBs have demonstrated superior environmental and economic performance compared to the more mature Vanadium Redox Flow Batteries (VRFBs).^20^ Further details on literature findings are discussed in the Results and Discussion section.

While the existing literature has primarily focused on maximizing RTE, it is imperative to address the Gross Energy Density (GED) indicator with equal attention, as it may hold greater relevance for decision-makers depending on the context. To illustrate, in scenarios where surplus renewable energy that would otherwise be “lost” (e.g., through the shutdown of photovoltaic panels or disconnection of wind turbines), it may be more advantageous to use this energy less efficiently (resulting in a lower RTE) if it enables the subsequent recovery of a larger amount of energy (resulting in a greater GED).

In this sense, the aim of this study is to enhance the knowledge of ABFBs through the direct experimental determination of key performance indicators, RTE and GED. To the best of the authors’ knowledge, it is the first time that GED has been reported for different charging and discharging current densities. Additionally, this study will present for the first time the possible trade-off between these two indicators, pointing out the difference among them.

## Materials and Methods

### Solutions Employed in the Study

The selection of the saline solution directly determines the composition of the acidic and alkaline solutions, as the chemistry of ABFBs is based on the added electrolyte.^21^ The selected electrolyte should be highly soluble at different pH values to prevent precipitations and scaling. Additionally, the electrolyte should be cheap and abundant and provide high conductivities in solution. Recently, Boulif et al.^12^ compared the performance of ABFB under different electrolytes, i.e., NaCl, KCl, and Na_2_SO_4_. Although all of these electrolytes enabled satisfactory operation of the ABFB, the presence of SO_4_^2−^ conferred greater cycling stability, albeit at the expense of reduced energy performance. Furthermore, it has been observed that salts comprising multivalent ions such as Mg^2+^, Ca^2+^, or Fe^2+/3+^ are not recommended due to their tendency to precipitate as hydroxides, leading to scaling on the surface of the membranes.^21,22^

In this study, NaCl has been used for ease of comparison with the existing literature. Accordingly, the acid and base solutions generated were HCl and NaOH, respectively. In particular, 1.0 M NaCl was prepared (99.5% Fisher Chemical, USA), whereas 0.1 M of both HCl (37% Fisher Chemical, USA) and NaOH (49−50% ACIdEKA, Spain) were used.

Due to its proven outstanding performance in reverse electrodialysis applications,^23,24^ the reversible redox pair [Fe­(CN)_6_]^3−^/[Fe­(CN)_6_]^4−^ was selected in order to minimize energy consumption during the charging phase and maximize energy recovery in the discharging phase. The ERS was composed by 0.05 M K_3_[Fe­(CN)_6_] (99% Scharlau, Spain), 0.05 M K_4_[Fe­(CN)_6_] (99% Scharlau, Spain), and 0.25 M NaCl (to increase conductivity) in acid media (HCl, pH <2.5) to avoid iron precipitation.

Milli-Q Integral system (Merck, Germany) grade II water was used to prepare all solutions.

### Lab-Scale Experimental Setup

Figure 2 depicts the layout of the lab-scale experimental setup utilized in this investigation.

**Figure 2:**
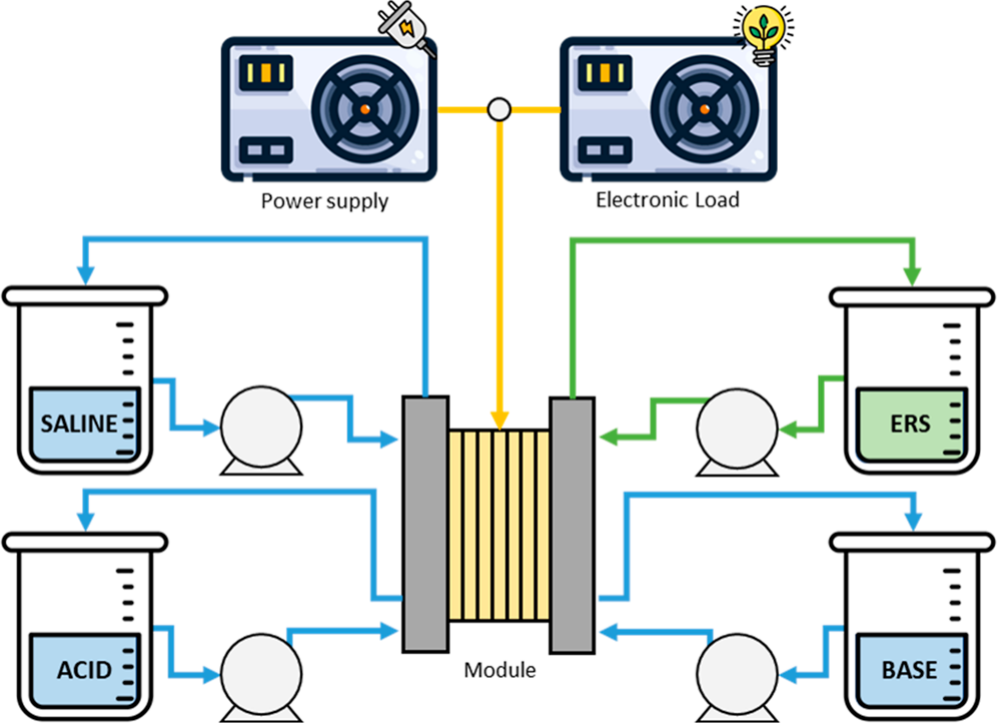
Lab-scale experimental setup.

The main element of the experimental setup is the BMED commercial module ED-3-100-10, purchased from Fumatech (Germany). The module is assembled with a stack of 10 triplets of commercial membranes (AEM: Fumasep FAB-130, CEM: Fumasep FKB-130, BPM: Fumasep FBM) from Fumatech (Germany), whose properties are summarized in Table 1.^25^ The specific area of each membrane is 100 cm^2^, and taking into account the 10 pairs of triplets, it provides a total of 1000 cm^2^ for each type of membrane (AEM, CEM, and BPM), amounting to a total of 3000 cm^2^. The membranes are separated by PVC/ECTFE spacers with a thickness of 500 μm, which create the compartments. The electrodes are iridium-coated titanium with an area of 100 cm^2^ (10 × 10 cm).

**Table 1:** Properties of the Membranes Used (According to the Manufacturer’s Datasheet)^25^

	membrane		
property	FAB-PK-130	FKB-PK-130	FBM
membrane type	AEM	CEM	BPM
reinforcement	PK	PK	PK
counter ion	Br^−^	H^+^	Na^+^ (CEM layer)/Cl^−^ (AEM layer)
thickness (dry) (μm)	110−150	120−150	110−160
area resistance (Ω·cm^2^)	<8.5^a^	<5.0^b^	
selectivity 0.1/0.5 mol/kg KCl at 25 °C^c^ (%)	>93	>98	
proton transfer rate (nmol/min/cm^2^)	<500^d^		
hydroxyl transfer rate (nmol/min/cm^2^)		<100^e^	
pH stability range at 25 °C (pH)	1−14	0−14	
water splitting voltage at 100 mA/cm^2^ ^f^ (V)			<1.2
water splitting efficiency at 100 mA/cm^2^ ^f^ (%)			>98

a In Cl^−^ form in 0.5 M NaCl at 25 °C measured in a standard measuring cell (through-plane).

b In Na^+^ form in 0.5 M NaCl at 25 °C measured in a standard measuring cell (through-plane).

c Determined from membrane potential measurement in a concentration cell.

d Determined from pH potential measurement in a concentration cell 0.1 M HCl/0.1 M NaCl at 25 °C.

e Determined from pH potential measurement in a concentration cell 0.5 M NaOH/0.5 M NaCl at 25 °C.

f In 0.5 M NaCl solution and 0.25 M Na_2_SO_4_ ERS at 25 °C.

The experimental setup also includes four tanks with a maximum capacity of 2 L each, containing the acid, base, saline solution, and ERS, which are pumped to the module by 323 Watson Marlow (UK) peristaltic pumps.

The experimental setup accounts for dead volumes of 150 mL in each of the acidic, alkaline, and saline solutions and 180 mL in the ERS solution, which include compartments within the module and tubing.

During the charging phase, electrical energy was applied by a laboratory power supply model 1580 from Peaktech (Germany). Although the experimental campaign has been carried out under a constant current density, equivalent results could be expected for a variable current density such as the one provided by renewable energy sources (e.g., solar photovoltaic or wind energy), as previously demonstrated in the literature.^6–8^

Conversely, in the discharging phase, an electronic load model Chroma 6312A (USA) was employed.

### Experimental Campaign and Procedure

In this study, ABFB performance was evaluated across a broad range of current densities during the charging phase (100−400 A/m^2^) and discharging phase (10−30 A/m^2^). It is noteworthy that current densities in the charging phase have been limited to ensure low energy consumptions, and a maximum of 30 A/m^2^ has been stablished for the discharging phase to prevent BPM delamination, as suggested by the literature.^5^ Specifically, six tests were conducted to allow for the independent evaluation of charging current density through the tests C100/20, C200/20, C300/20, and C400/20; and the independent evaluation of discharging current density through the tests C100/10, C100/20, and C100/30. In the test codification, “C” denotes the cycle number, while the accompanying numbers indicate the fixed current densities (in A/m^2^) applied during the charging and discharging phases, respectively.

All tests are comprised of two discrete phases, starting from the charging phase and subsequently followed by the discharging phase.

In the initial stage of the experiment, the acid and base tanks were filled with 1 L of 0.1 M HCl and NaOH solutions, respectively. The saline solution was prepared at a concentration of 1.0 M NaCl to a volume of 1 L. Two liters of ERS (composition previously described) were employed. To prevent channel clogging, which could result in overpressure, all solutions are filtered. Each solution loop is pumped to the module at a flow rate of 1000 mL/min (linear velocity of ∼3.5 cm/s). Once the module is filled and air bubbles are removed, the charging phase starts by turning on the power supply and setting the current to provide the desired current density value (galvanostatic operating mode). The duration of the charging phase was determined in preliminary tests conducted in our laboratory as a function of the applied current density. These operating times ensure the maximization of product concentration (i.e., increasing the operating time would not result on higher concentrations) without completely depleting the saline feed, thus avoiding overpotentials and preventing a significant increase in energy consumption through the extension of this phase. Upon completion of the charging phase, the power supply is disconnected, and the volumes of the solutions are measured in order to quantify the electro-osmotic and osmotic water transport as well as to detect any potential anomalies such as leakages between compartments.

The discharge phase begins with the solutions derived from the charging phase, specifically concentrated acids and bases and diluted saline solution. Once the solutions are in circulation, the electronic load is connected with the current set to the selected discharge current density (galvanostatic operating mode). The discharging phase is maintained until either the voltage drops to zero or the current falls below the specified fixed value. Upon completion of the discharging phase, the electronic load is disconnected, the module is emptied, the pumps are turned off, and the volumes of the solutions are measured. After that, the module is cleaned by circulation of deionized water in an open loop to avoid salt accumulation in the compartments and tubes.

The voltage and current were monitored through displays of the power supply or electronic load.

Moreover, periodic leakage tests are conducted as well as the replication of a test to verify the reproducibility and proper performance of the system.

### Analytical Methods

Furthermore, samples were periodically withdrawn with the purpose of measuring the pH, conductivity, temperature, and concentrations of H^+^ and OH^−^ ions in the acid and base solutions, respectively. The pH and the temperature were measured using a pH/Temperature Bench Meter Mi 150 (Milwaukee, USA). Conductivity measurements were conducted using a CLS50D probe (Endress + Hauser, Germany). The concentrations of H^+^ in the acid and OH^−^ in the base were determined through titration, using commercial 0.1 N NaOH and HCl solutions (Fisher Chemical, USA) and phenolphthalein (1% solution in ethanol, Scharlau, Spain) as the indicator. Each titration was carried out in triplicate to ensure accuracy, precision, and reliability of results.

### Calculation of Performance Indicators

This study assesses the performance of an ABFB through the Round-Trip Efficiency (RTE) and Gross Energy Density (GED).

RTE is defined as the ratio between the energy released during the discharge phase and the energy supplied during the charging phase (eq 1):

1 $$RTE\;\left(\%\right)=\frac{\int_{0}^{t_{d}}{I_{d}\times V}_{d}\;dt}{\int_{0}^{t_{c}}{I_{c}\times V}_{c}\;dt}\times 100$$

where *t* is the time (h), *I* represents the current (A), *V* denotes the voltage (V), and the subscripts d and c refer to the discharge and charge phases, respectively.

The GED (Wh/L) is a measure of the physical size of the system required to store a given amount of energy. This relationship is reflected in eq 2, which demonstrates the ratio between the energy recovered during discharge and the volume of the acidic solution at the beginning of the discharge:

2 $$GED\;\left(Wh/L\right)=\frac{\int_{0}^{t_{d}}{I_{d}\times V}_{d}\;dt}{{Vol}_{HCl}}\times 100$$

where *t* is the time (h), *I* represents the current (A), *V* is the voltage (V), the subscript *d* refers to the discharge phase, and Vol_HCl_ is the volume of acid solution at the beginning of the discharge.

## Results and Discussion

### Concentration Profiles in Charging−Discharging Experiments

The concentrations of both NaOH and HCl exhibit comparable behavior; therefore, Figure 3 illustrates the evolution of the average acid and base concentrations along charging and discharging phases for all the tests conducted. Further details on the specific H^+^ concentration in the acid and OH^−^ in the base can be found in Figure S1 of the Supporting Information. In all trials, a peak concentration is observed that coincides with the end of the charging phase and the initiation of the discharging phase. The peak concentration is emphasized in Figure 3 through the displayed data levels. Additionally, salt concentration, assessed via conductivity, evidences an inverse trend (reduction-increase), as reported in Figure S2 of the Supporting Information.

**Figure 3:**
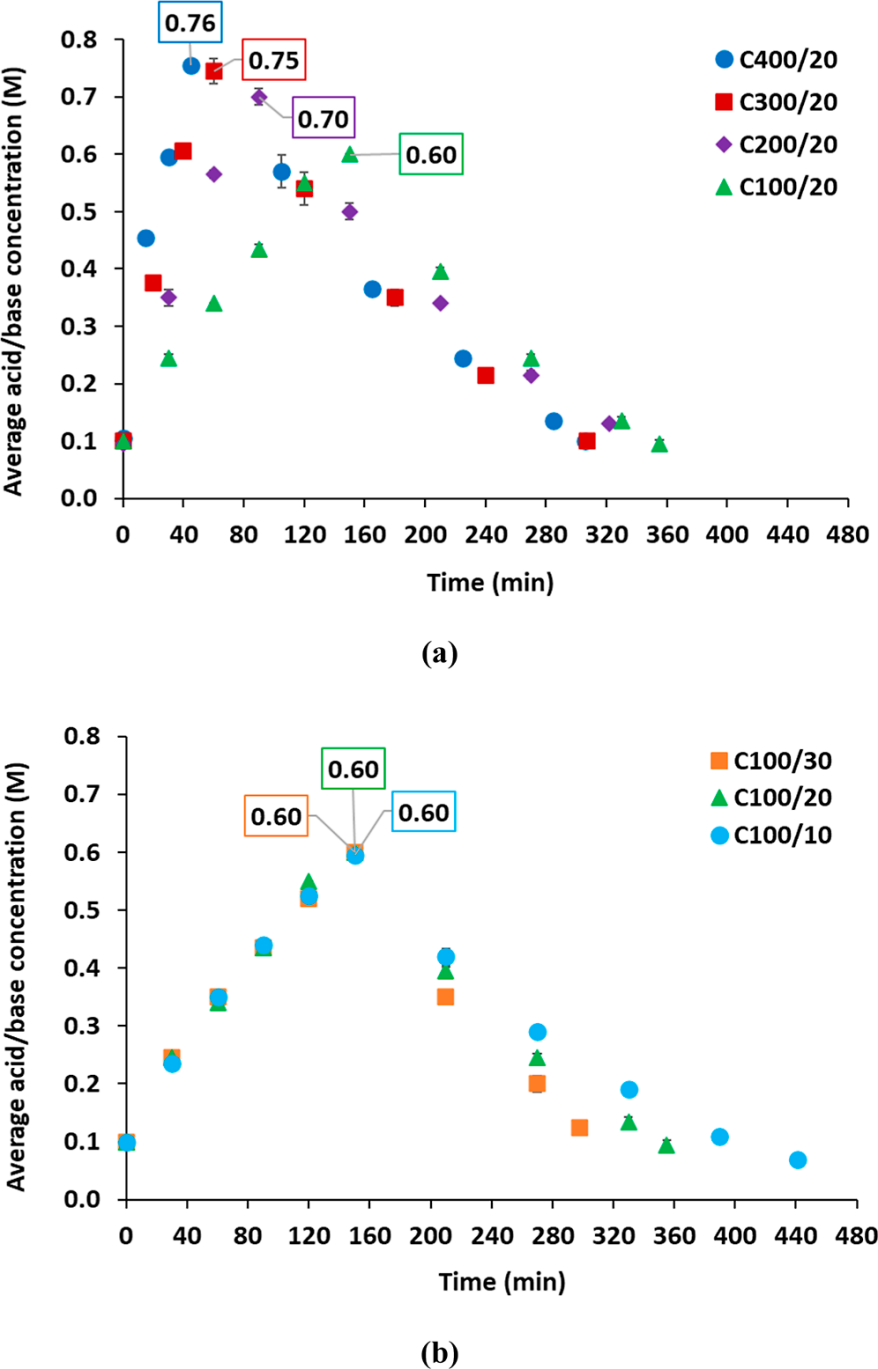
Evolution of average acid and base concentrations through the ABFB performed tests. Influence of: (a) the charging current density (at a fixed discharging current density of 20 A/m^2^) and (b) the discharging current density (at a fixed charging current density of 100 A/m^2^).

Besides, it must be pointed out that the comparison of total initial and final solution volumes, accounting for the volumes withdrawn for sampling, showed discrepancies below 2%, ensuring proper performances. These differences can be attributed to minor residual volumes remaining in the module or tubing due to the practical limitations of complete drainage.

As expected, the application of higher charging current densities has been observed to result in higher acid and base concentrations, concomitant with a decrease in the requisite times. It should be noted that the concentration of the products is limited by two factors: first, the applied current density and, second, the concentration and volume of the saline solution (NaCl). Consequently, even if the operation times were extended, the concentrations of the products would remain constrained.

As illustrated in Figure 3a, a charging current density of 400 A/m^2^ results in the attainment of average concentrations of 0.76 M for HCl and NaOH. This is in contrast with the maximum concentration of 0.60 M obtained at 100 A/m^2^, despite the saline solution remaining in a nondepleted state. In this instance, the nonideal phenomena such as the diffusive transport of ions (in accordance with the concentration gradient) become evident, thereby counteracting the electromigratory transport. On the other hand, Figure 3b presents the results for the charging phase conducted under the same conditions, demonstrating the good reproducibility of the experiments.

The aim of traditional BMED studies is to achieve the greatest possible concentration of products. This is accomplished through the implementation of strategies such as the utilization of high current densities, the oversizing of saline solution volumes to prevent a reduction in its concentration, or even the use of higher saline concentrations.^8,26^ However, these strategies are not applicable during the ABFB charging phase as they would result in a reduction in performance due to two main factors: (a) increased energy consumption, which is caused by resulting from higher current densities; and/or (b) decreased energy recovery, which is caused by elevated concentrations in the saline channel.

For the future implementation of ABFBs in a real-world setting, it is useful to understand the charging process and discharging process operating times as well as the relationship between them. Extended charging times and shortened discharging times may restrict the applicability of any type of battery. Hence, Figure 4 illustrates the charging and discharging times, as well as the total operating time. The operating time of the discharge phase is determined by two factors: (a) the concentration reached during the charging phase and (b) the discharging current density selected. Larger charging current densities and consequently, higher HCl and NaOH concentrations, increase the pH gradient, leading to longer operating times in the discharging phase (Figure 4a). In this study, a range of 205 to 261 min was obtained when charging current densities of 100 to 400 A/m^2^ were employed, respectively. Conversely, the utilization of higher discharging current densities shortens the discharging operating time, thereby facilitating a more rapid decrease in the pH gradient due to the accelerated neutralization process (Figure 4b). In this study, a decrease from 291 to 148 min was observed when discharging current densities were increased from 10 A/m^2^ to 30 A/m^2^. Additionally, the utilization of lower discharge current densities allows a more effective usage of the pH gradient as less energy is required to provide the set discharging current density. This fact enlarges discharging operating times (Figure 4b) due to the neutralization until lower concentrations of HCl and NaOH are at the end of the discharging phase. This is illustrated in Figure 4b, which depicts the variation in acid and base concentrations, which range from 0.07 to 0.13 M, when the discharging current density was increased from 10 A/m^2^ to 30 A/m^2^.

**Figure 4:**
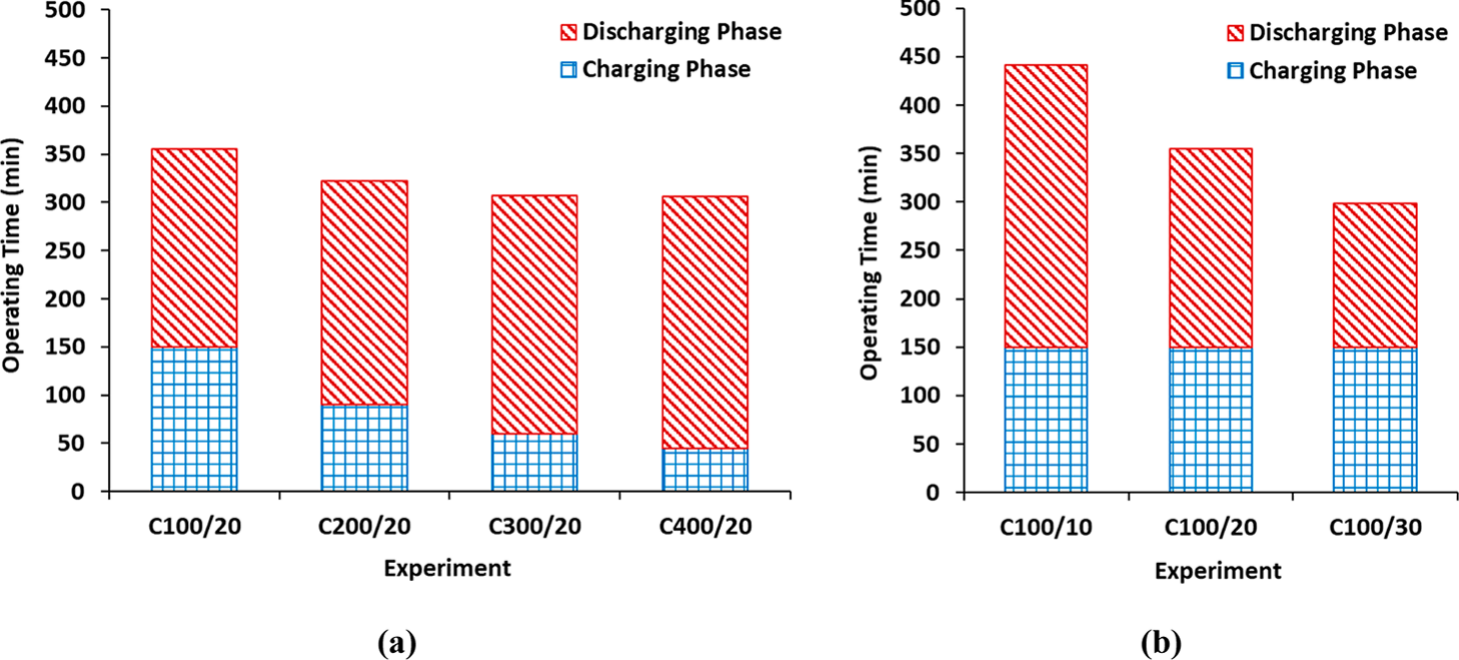
Comparison of operating time of the charging phase, the discharging phase, and the total cycle: (a) charging current density (at a fixed discharging current density of 20 A/m^2^) and (b) discharging current density (at a fixed charging current density of 100 A/m^2^).

### Round-Trip Efficiency Results

Monitoring the voltage profiles (see Figure S3 in the Supporting Information) over the charging and discharging phases enabled a detailed assessment of the energy consumed during the charging stage and that recovered during the discharging stage.

The RTE has been determined according to eq 1, and Figure 5 displays the corresponding results for each experiment. It can be observed that the results are maximized when low current densities are used during charging (Figure 5a) and when high current densities are used during discharging (Figure 5b). The C100/30 experiment yielded a maximum RTE value of 12.6%, which corresponds to a charging current density of 100 A/m^2^ and a discharging current density of 30 A/m^2^. While reducing the charging current density or increasing the discharging current density could potentially improve the RTE, this could also have an adverse impact on the ABFB performance due to insufficient HCl and NaOH concentrations and the potential for BPM delamination.

**Figure 5:**
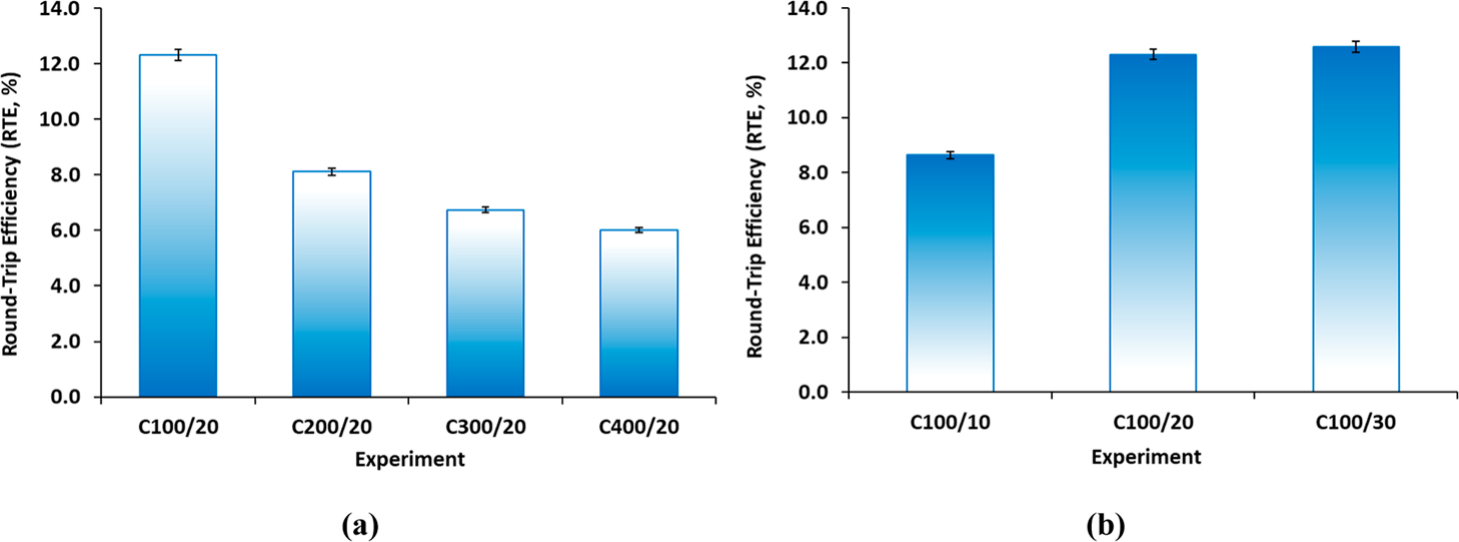
RTE results of the ABFB performed tests. Evaluation of: (a) the charging current density (at a fixed discharging current density of 20 A/m^2^) and (b) the discharging current density (at a fixed charging current density of 100 A/m^2^).

These seemingly low RTE values may appear unattractive for conventional battery applications where they would imply significant energy losses. However, ABFBs, known for their versatility and fast response to dynamic conditions, exhibit a strong potential for applications involving the capture of renewable energy production peaks. These peaks often exceed the grid’s absorption capacity and may otherwise be wasted or even result in the disconnection of generation systems such as photovoltaic panels or wind turbines.

As expected, the existing literature consistently aims to maximize the RTE of ABFBs to enhance their appeal to stakeholders. In this context, mathematical modeling studies have been undertaken to simulate and optimize ABFB system performance.^27,28^ A summary of literature findings can be found in Table S1 of the Supporting Information. For example, Culcasi et al.^27^ investigated, through simulations, the impact of module geometry and membrane properties on RTE. Their findings showed that reducing the manifold diameter from 6 mm to 2 mm increased RTE from 17% to 40%, claiming a reduction of parasitic currents. Similarly, an RTE of 64% was predicted for a spacer thickness of 278 μm, although this remains below typical commercial values (e.g., 500 μm for the Fumatech lab-scale ED-3-100 stack). Although these results are promising from an RTE perspective, geometric modifications must be approached with caution, as they can negatively affect hydrodynamic behavior by increasing pressure drops or causing uneven flow and solution distribution within the channels. In the same study, a simulated RTE of 76% was achieved by halving key membrane properties, including electrical resistance, ion diffusivity, and water permeability, in the simulations. Despite the fact that these proposed configurations remain distant from the current state-of-the-art, the corresponding RTE values can be considered as ideal targets for future technological developments.

The same research group has recently investigated, through experimental methods, the effect of employing manifold section reducers on the performance of ABFBs.^16^ The same module and membranes as those used in this study were employed. In contrast, 30 triplets were assembled, AEMs were used as end membranes (FeCl_2_/FeCl_3_ was the ERS), and a flow velocity of 0.2 cm/s was selected. This approach led to an increase in RTE from 2.6% to 9.2% through the use of manifold reducers at a charging:discharging current density ratio of 100:29 A/m^2^. The utilization of manifold reducers enabled the attainment of a product concentration that was twice that of the original. The RTE value reported by Pellegrino et al.^16^ has been surpassed in the current study, with the following possible reasons: (a) the use of a larger flow velocity, which minimizes the influence of a boundary layer on ion transport across membranes (the manufacturer recommends flow velocities up to 10 cm/s); (b) energy losses associated with an increased number of triplets, which reduce the open-circuit voltage (OCV) per triplet (nonideal phenomena become more pronounced), while simultaneously increasing the electrical resistance; and (c) the use of different ERS solution and end-membranes, which may impact the electrical resistance associated with the electrode compartment (also known as blank resistance).

Furthermore, the establishment of clear and standardized methodologies for calculating indicators, such as RTE, is necessary to ensure reliable and equitable comparisons. Al-Dhubhani et al.^29^ reported an impressive RTE value of 65% for a discharge-to-charge current density ratio of 60:240 A/m^2^ in a single-cell ABFB. This value was attained by the authors as a result of employing an alternative methodology for RTE calculation, namely, as the product of Voltage Efficiency (VE, ratio of the integral of the voltage during discharge and the integral of the voltage during charge) and Coulombic Efficiency (CE, ratio of the integral of the current during discharge and the integral of the current during charge). This approach, which differs from the one outlined in eq 2, is mathematically correct only when charging and discharging times are equal. Nevertheless, when RTE is calculated through eq 2, the value is reduced to 25%. Despite this result being more promising than the one reported in this work (12.6%), it must be taken into account that (a) a greater number of triplets are employed (10 vs 1), which is closer to a real application scenario; and (b) the use of a discharging current density of 60 A/m^2^ could potentially result in BPM delamination if long discharging operating times are involved.

### Gross Energy Density Results

In contrast to RTE, GED is seldom the subject of detailed analysis in the literature. Instead, reporting is typically limited to the theoretical value or to predicted values derived from polarization curves, but examples of direct experimental results are scarce. This study examines, calculates, and provides the value of GED under different charging and discharging current densities. Figure 6 presents the GED values calculated according to eq 2. Higher pH gradients, resulting from increased charging current densities, led to elevated GED values (Figure 6a). Likewise, elevated discharging currents contributed to higher GED (Figure 6b). In this context, a maximum value of 4.2 Wh/L HCl was obtained in the C400/20 test, corresponding to a charging current density of 400 A/m^2^ and a discharging current density of 20 A/m^2^. A comparison between the results of C100/20 and C100/30 (Figure 6b) reveals that the discharging current density exhibits a minimal impact, with the most significant variations occurring during the charging phase. Therefore, it is hypothesized that the combination of charging:discharging current densities of 400:30 A/m^2^ may result in a slight increase in GED.

**Figure 6:**
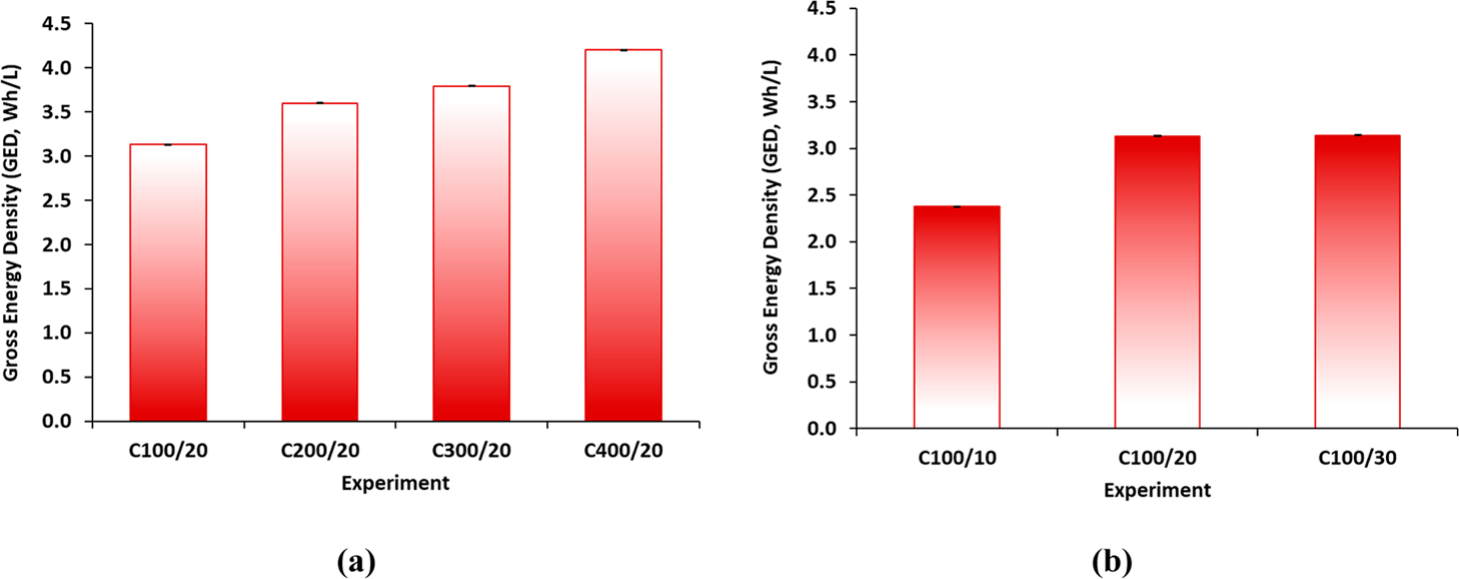
GED results in the ABFB performed tests. Evaluation of: (a) the charging current density (at a fixed discharging current density of 20 A/m^2^) and (b) the discharging current density (at a fixed charging current density of 100 A/m^2^).

As previously outlined, the extant literature offers a range of estimations and predictions for GED. However, a summary of literature findings can be found in Table S1 of the Supporting Information. The theoretical thermodynamical GED of 1 M HCl–NaOH neutralization is 24 Wh/L (normalized on the volume of one feed solution, typically the acid).^10,21^ Van Egmond et al. obtained a value of 2.9 Wh/L on a single cell device.^10^ Zaffora et al.^14^ predicted a value of 10.3 Wh/L based on the polarization curves of the discharging phase from 1 M HCl and NaOH and a 100 A/m^2^ (not technically feasible at the current state of the art of BPM due to delamination phenomenon). Pärnamäe et al.^21^ conducted a mathematical simulation to maximize the cumulative GED from 10.4 Wh/L to 17.4 Wh/L increasing the number of stages in series from 4 to 17.

In this respect, the findings presented in this study, ranging from 2.4 to 4.2 Wh/L were derived directly from the ABFB experiments, as opposed to being estimated or mathematically simulated.

### Trade-Off between Round-Trip Efficiency and Gross Energy Density

The RTE and GED data previously presented allow us to conclude that the operating conditions, specifically the discharging current density, that maximize RTE and GED are opposite. In order to achieve the greatest RTE, low charging current densities (100 A/m^2^ in this study) are preferred. Conversely, high charging current densities (400 A/m^2^ in this study) are preferred for maximizing GED. To better illustrate this point and assist decision-makers in making trade-off decisions, Figure 7 presents the relationship between RTE and GED under the experimental conditions considered in this study. The closest point to the upper right corner represents the conditions under which both performance indicators (RTE and GED) are maximized simultaneously. Among the studied conditions, the C100/30 experiment appears to present the most optimal operating conditions, with a RTE value of 12.6% and a GED value of 3.1 Wh/L. Nevertheless, almost the same results of RTE and GED were obtained for the C100/20, with values of 12.3% and 3.1 Wh/L, respectively. Although increasing the discharging current density favors both RTE and GED, the current development of BPMs limited this increment, as can be seen by comparison of the results of C100/30 and C100/20.

**Figure 7:**
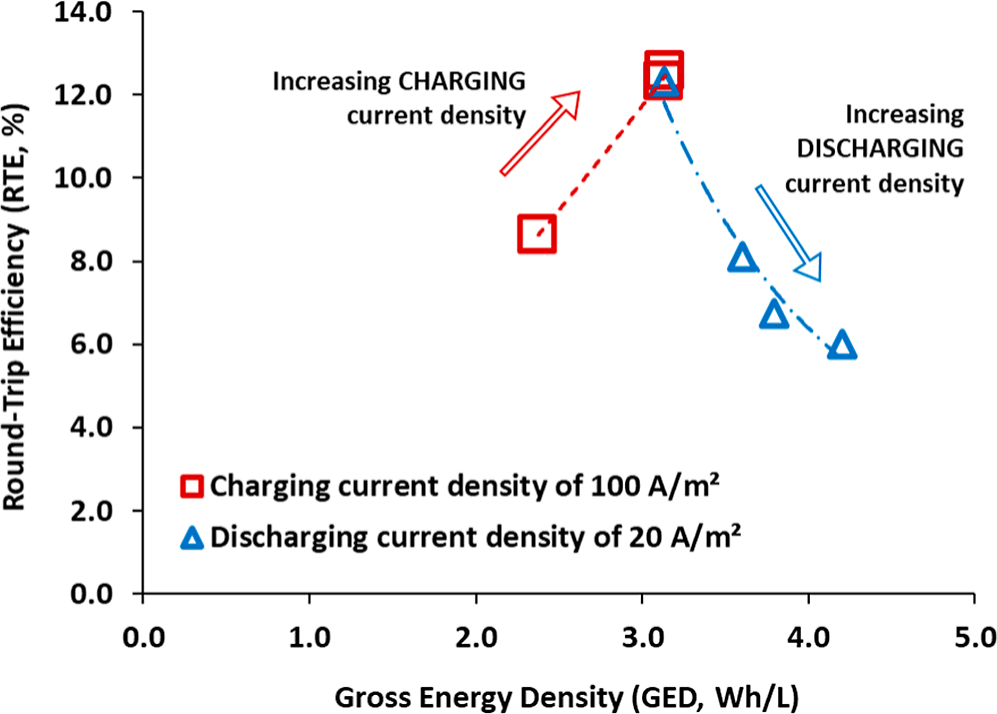
Relation between RTE and GED in the performed experiments.

However, it should be noted that there may be scenarios in which maximizing GED is a priority, even though it may penalize the RTE. In such cases, higher charging current densities should be selected, as shown in blue in Figure 7. For instance, experiment C400/20 maximizes GED with a value of 4.2 Wh/L by decreasing the RTE to 6.0%. A clear example of prioritizing GED over RTE is the utilization of renewable energy surges that would otherwise be ″lost″ due to the unavailability of equipment capable of operating under conditions that maximize RTE. Such circumstances may result in the shutdown of equipment, such as photovoltaic panels or wind turbines. In such cases, it may be more advantageous to consume the energy less efficiently during the ABFB charging phase, resulting in higher energy consumption, in order to achieve a greater energy return during the discharging phase.

Similarly, from an economic perspective, it may be desirable to consume more energy when its price is low, even at the expense of RTE, with the objective of recovering a greater amount of energy when its price is higher.

The outcomes yielded for both RTE and GED, and subsequently their trade-off, are technically constrained by the current developmental stage of the devices, including both the modules and the membranes that configure the stack. The development of membranes with lower electrical resistance would permit a reduction of the electrical consumption during the charging process and an increment in the energy recovery during the discharging process. The development of BPMs with a focus on energy recovery applications would significantly enhance the outcomes by enabling higher current densities during discharging.

## Conclusions

This work presents advances in the open literature about the combination of BMED and BMRED within ABFB systems, focusing on the performance evaluation of ABFB in terms of both RTE and GED, as well as the analysis of their trade-offs based on experimental results.

Under the experimental conditions employed, the RTE is maximized by using low charging current densities; despite limiting the product concentrations obtained, a maximum RTE of 12.6% was achieved with a charge-to-discharge current density ratio of 100:30 A/m^2^. Conversely, GED is maximized when the concentration of acid and base achieved during charging is highest. GED is also maximized by applying high discharging current densities. Among the operating conditions studied, a maximum GED of 4.2 Wh/L was achieved with a charge-to-discharge current density ratio of 400:20 A/m^2^.

Thus, under the operating conditions studied, it is not possible to maximize both the RTE and GED simultaneously. Although traditionally the selected indicator has been RTE, GED may be prioritized in certain scenarios, even at the expense of RTE. This approach could be particularly advantageous when harnessing renewable energy surpluses that would otherwise go unused or during periods when energy prices are extremely low.

Future work focused on improving membrane properties, such as reducing electrical resistance or enhancing BPM mechanical resistance to delamination, along with better module design, could boost the ABFB performance.

## Supplementary Material



## Notes

Evolution of H^+^ in the acidic solution and OH^−^ in the basic solution, as well as the conductivity of the saline solution. Voltage evolution profiles. Summary of literature works reporting RTE and GED values (PDF)

## Abbreviations

ABFB: acid−base flow battery
AEM: anion exchange membrane
BMED: bipolar membranes electrodialysis
BPM: bipolar membrane
CE: Coulombic efficiency
CEM: cation exchange membrane
ERS: electrode rinse solution
GED: gross energy density
OCV: open-circuit voltage
BMRED: bipolar membrane reverse electrodialysis
RTE: round-trip efficiency
VE: voltage efficiency
VRFB: vanadium redox flow battery
