# Supplementary material for: Acid−Base Flow Batteries for Sustainable Energy Storage: Balancing Energy Recovery and Efficiency
Source: ACS Omega. 2025 Nov 28;10(48):58420–8. doi: 10.1021/acsomega.5c06024 (PMC12771209; doi:10.1021/acsomega.5c06024)
Supplement: Supplementary file 1 [file ao5c06024_si_001.pdf]

Supporting Information - Acid-Base Flow Batteries (ABFBs) for Sustainable Energy Storage: Balancing Energy Recovery and Efficiency

Marta Herrero-Gonzalez[a,*], Nadia El Arroubi[a], María Fresnedo San-Roman[a], Raquel Ibañez[a]

[a] *Chemical and Biomolecular Engineering Department, Universidad de Cantabria, Avda. Los Castros 46, Santander, 39005, Spain*

[*]Corresponding author.

E-mail address: herrerogma@unican.es (M. Herrero-Gonzalez)

Acid and base concentration evolution

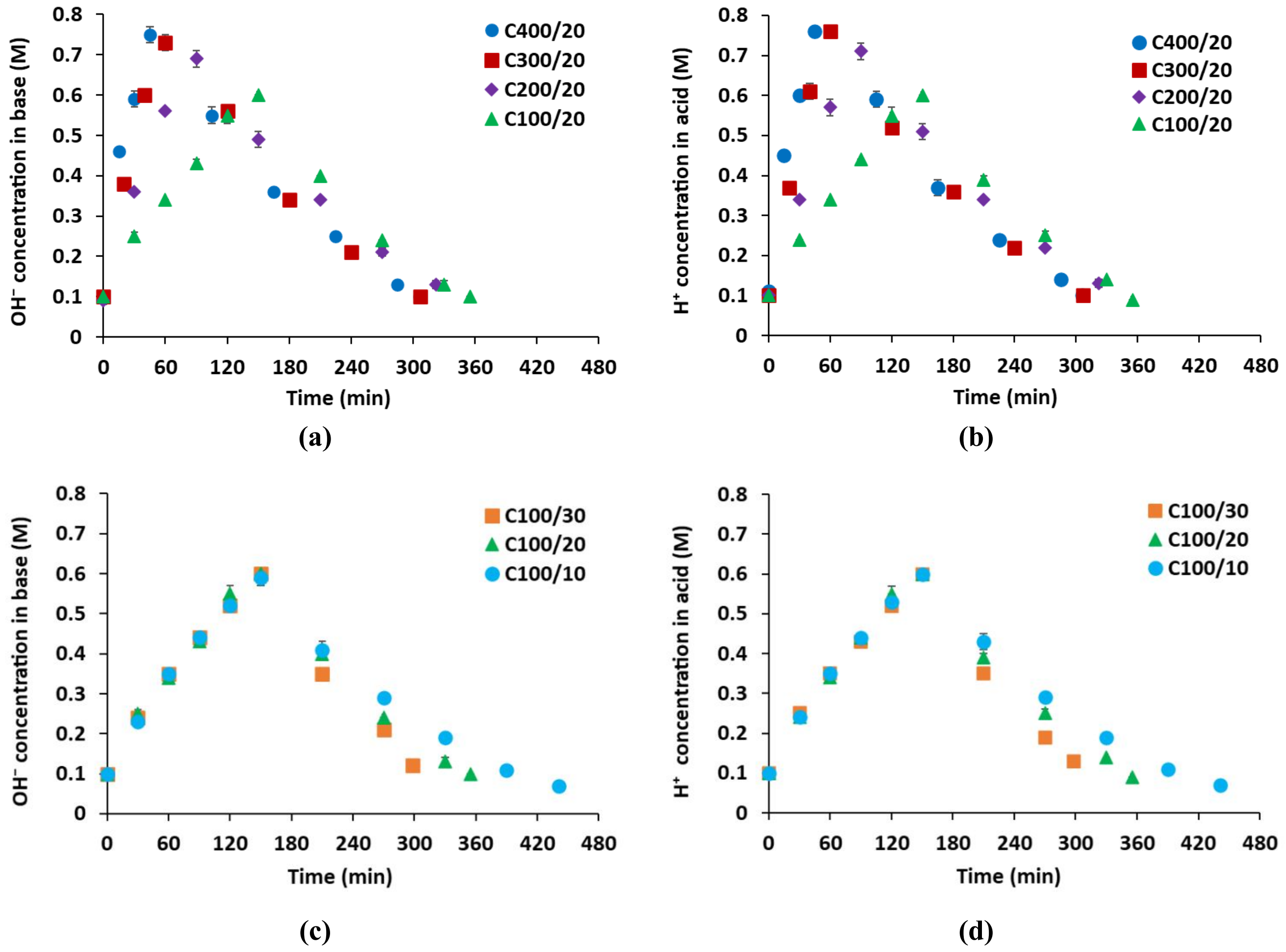

Figure S1. Evolution of: a) OH^- concentration of the base at a discharging current density of 20 A/m^2, b) OH^- concentration of the base at a charging current density of 100 A/m^2, c) H^+

concentration of the acid at a discharging current density of 20 A/m², and d) H^+ concentration of the acid at a charging current density of 100 A/m².

Saline solution conductivity evolution

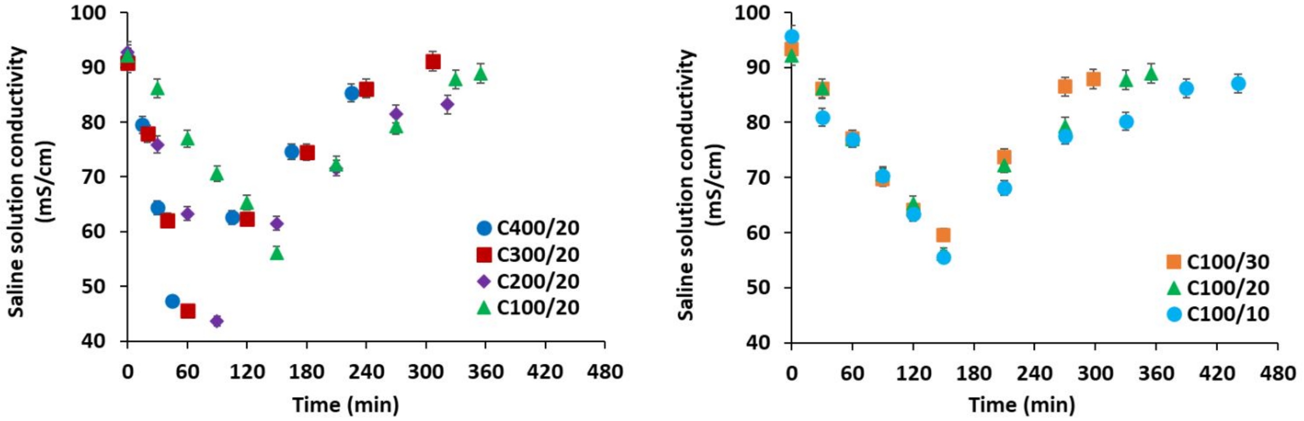

(a) (b)

Figure S2. Evolution of: a) saline conductivity at a discharging current density of 20 A/m², and b) saline conductivity at a charging current density of 100 A/m².

Voltage evolution

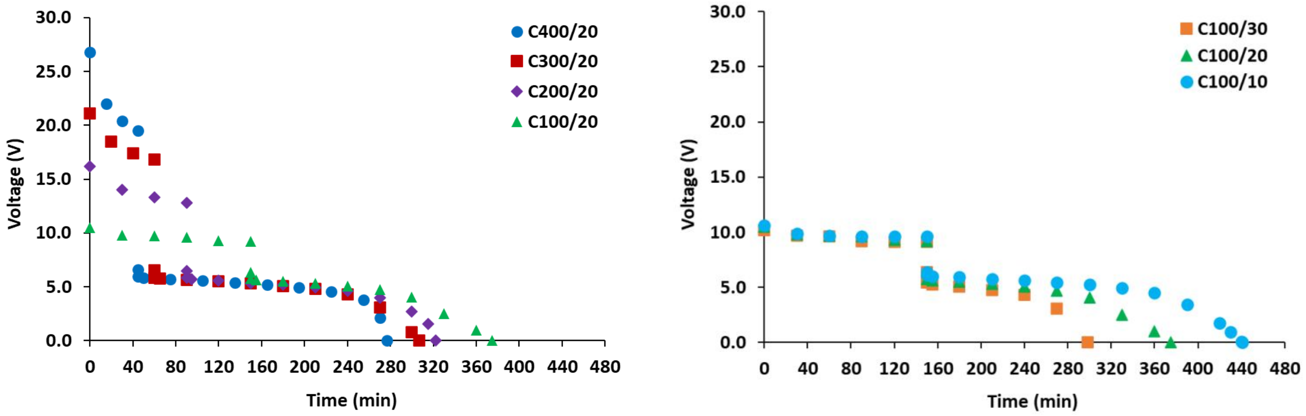

(a) (b)

Figure S3. Evolution of voltage in: a) test at a discharging current density of 20 A/m², and b) test at a charging current density of 100 A/m².

Summary of literature works reporting RTE and GED values

Table S1. Summary of literature works reporting RTE and GED values

Ref.	Phase/s studied	Type of study	Focus of the study	Stack size	Membranes	RTE	GED
Van Egmond et al., 2018[1]	Both	Experimental	Charging/discharging current densities (50, 100, 150 A/m^2 / 5, 10, 15 A/m^2)	Single cell Area 100 cm^2	FAB-PK-130, FBM from Fumatech (Germany) Nafion N117 Dupont (USA)	Up to 13.5% Reported for different conditions	Up to 2.9 Wh/L Not reported for different conditions
Zaffora et al. 2020[2]	Discharging phase	Experimental	Influence of operating conditions (flow velocity, number of triplets, background salt) on polarization curves under different pH gradients	5, 10, 38 triplets Area 100 cm^2	FAB-PK-130, FKB-PK-130, FBM from Fumatech (Germany)	Not applicable	10.3, value extrapolated from polarization curve at discharging current density of 100 A/m2
Pellegrino et al. 2024[3]	Both	Experimental and modeling	Experimental: Use of manifold reducers Modeling: optimized design features	30 triplets Area 100 cm^2	FAB-PK-130, FKB-PK-130, FBM from Fumatech (Germany)	Experimental: up to 9.2% Modelled: up to 64% with optimized stack design and membranes not available	Not reported
Culcasi et al. 2023[4]	Both	Modeling	Optimization by variations in operating parameters and stack desing			Up to 40% by reducing manifolds Up to 64% by reducing spacer thickness Up to 76% by halving membrane properties (i.e. electrical resistance)	Not reported
Al-Dhubhani et al. 2020[5]	Both	Experimental	Different discharging to charging current density ratios	Single cell	FAB-PK-130, FKB-PK-75, FBM from Fumatech (Germany)	Up to 65% at 60:240 A/m^2 Calculations do not meet the expression of this work, when corrected, values up to 25%	Not reported
Pärnamäe et al. 2020[6]	Both	Modelling	Addition of multiple staks or stages			Based on results from [7] RTE 31–63% by increasing the number of stages	GED 10.4-17.4 Wh/L by increasing the number of stages Not reported under different operating conditions

References

(1) van Egmond, W. J.; Saakes, M.; Noor, I.; Porada, S.; Buisman, C. J. N.; Hamelers, H. V. M. Performance of an Environmentally Benign Acid Base Flow Battery at High Energy Density. *Int J Energy Res* 2018, *42* (4), 1524–1535. https://doi.org/10.1002/er.3941.

(2) Zaffora, A.; Culcasi, A.; Gurreri, L.; Cosenza, A.; Tamburini, A.; Santamaria, M.; Micale, G. Energy Harvesting by Waste Acid/Base Neutralization via Bipolar Membrane Reverse Electrodialysis. *Energies (Basel)* 2020, *13* (20). https://doi.org/10.3390/en13205510.

(3) Pellegrino, A.; Culcasi, A.; Cosenza, A.; Cipollina, A.; Tamburini, A.; Micale, G. Reducing Parasitic Currents in Acid-Base Flow Batteries by Decreasing the Manifold Cross-Sectional Area: Experiments and Modelling. *Chem Eng Sci* 2024, *299*, 120438. https://doi.org/10.1016/j.ces.2024.120438.

(4) Culcasi, A.; Gurreri, L.; Tamburini, A.; Cipollina, A.; Bogle, I. D. L.; Micale, G. Improving Efficiency and Discharge Power of Acid-Base Flow Battery via a Bi-Objective Optimisation. *J Energy Storage* 2023, *66*, 107429. https://doi.org/10.1016/j.est.2023.107429.

(5) Al-Dhubhani, E.; Pärnamäe, R.; Post, J. W.; Saakes, M.; Tedesco, M. Performance of Five Commercial Bipolar Membranes under Forward and Reverse Bias Conditions for Acid-Base Flow Battery Applications. *J Memb Sci* 2021, *640*, 119748. https://doi.org/10.1016/j.memsci.2021.119748.

(6) Pärnamäe, R.; Gurreri, L.; Post, J.; van Egmond, W. J.; Culcasi, A.; Saakes, M.; Cen, J.; Goosen, E.; Tamburini, A.; Vermaas, D. A.; Tedesco, M. The Acid–Base Flow Battery: Sustainable Energy Storage via Reversible Water Dissociation with Bipolar Membranes. *Membranes (Basel)* 2020, *10* (12), 1–20. https://doi.org/10.3390/membranes10120409.

(7) Culcasi, A.; Gurreri, L.; Zaffora, A.; Cosenza, A.; Tamburini, A.; Micale, G. On the Modelling of an Acid/Base Flow Battery: An Innovative Electrical Energy Storage Device Based on PH and Salinity Gradients. *Appl Energy* 2020, *277*, 115576. https://doi.org/10.1016/j.apenergy.2020.115576.
